# Unforeseen cascading effects of an inlet opening

**DOI:** 10.1038/s41598-024-63467-0

**Published:** 2024-06-11

**Authors:** Óscar Ferreira

**Affiliations:** CIMA-Centre for Marine and Environmental Research, FCT, University of Algarve, Faro, Portugal

**Keywords:** Inlet opening, Tidal channel, Coastal retreat, Ria Formosa, Environmental sciences, Natural hazards, Ocean sciences

## Abstract

The opening of the Cacela Inlet (southern Portugal) in 2010 led to unforeseen effects observed after 2017, including an extreme acceleration of the retreat of the inland lagoon margin from about 0.2 to 2 m/year. This was a consequence of the development of a large flood delta in an area of limited accommodation space, forcing the main tidal channel to move inland. The coastal retreat currently affects a flat sandy area that separates the old and inactive Cacela cliff from the lagoon. Between 2035 and 2040, the currently inactive Cacela cliff is likely to become active again, posing a potential risk of damage to a medieval fortress and the existing settlement of Cacela Velha, an unforeseen cascading effect of the opening of the inlet. In order to prevent instability and damage to this legally protected area of national and public interest, several coastal management measures will be required.

## Introduction

Tidal inlets play a crucial role in facilitating the exchange of water, nutrients, and sediments between the backbarrier lagoon and the adjacent open sea on barrier systems^1^. They are also a significant and dynamic component of such systems, with many exhibiting migratory behaviour. The openness of tidal inlets within a particular location is dependent on the relative influence of forcing conditions (waves and tides) and sediment availability. To maintain the efficiency of the water exchange, inlet opening, relocation, or dredging may be necessary^2,3^. Therefore, inlet relocation and new inlet opening are common coastal management approaches for barrier systems.

The relocation or artificial opening of an inlet can significantly alter the morphology of the nearby nearshore and backbarrier areas, as well as the shoreline position both updrift and downdrift. The changes in morphology near the new inlet will depend on the relationships between longshore transport, waves, and tidal currents and can occur over many years or even decades, often extending for kilometers along the coast^4,5^. After opening, the inlet acts as a barrier to longshore sediment transport and as a sediment trap^6–8^. The retained sediments will be incorporated in ebb and flood tidal deltas, recurved spits, or in shoals and banks^7^. These changes in sediment transport and deposition patterns also promote accretion at the updrift area and erosion downdrift^5,7,9^. The morphological behaviour following inlet opening has been extensively documented in several studies, with the magnitude of the transformations varying from site to site. However, a new inlet opening can lead to direct or indirect morphological and morphodynamic consequences that are not easy to anticipate. Some reported unforeseen consequences include: the forcing of the main tidal channel migration towards the new inlet position^10^; the inlet migration in the opposite direction to that historically observed^11,12^; the reduction of section and tidal prism at a nearby inlet leading to ebb delta attachment and consequent island growth for more than seven decades^2,3,13^; occurrence of beach erosion both updrift and downdrift of the new inlet associated to the growth of the ebb tidal shoal^14^; increased water levels and unexpected changes in hydrology with morphological repercussions^14,15^; and increased vulnerability of the lagoon ecosystem^16^.

**Figure 1:**
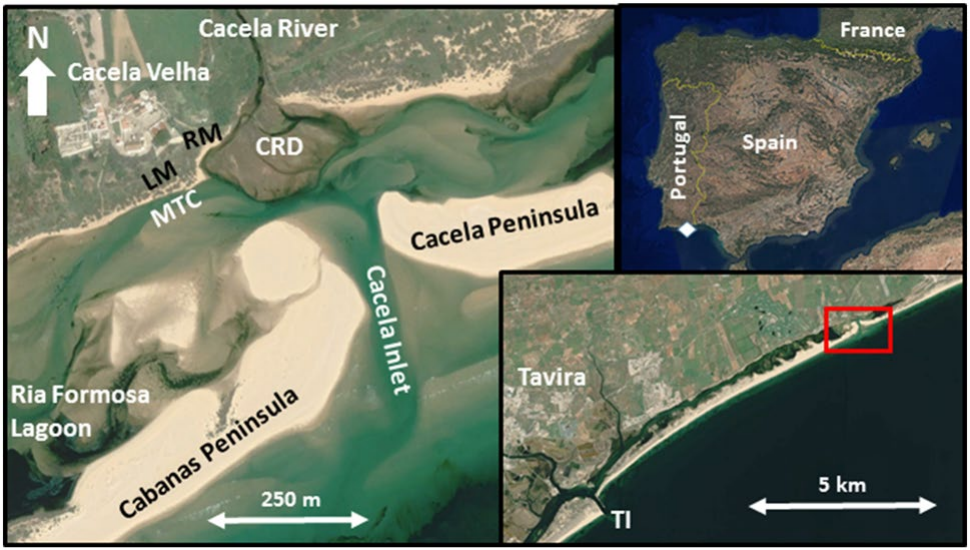
Location of the study area, including the main toponyms and morphologies: TI (Tavira Inlet), MTC (Main Tidal Channel), CRD (Cacela River Delta), LM (Lagoon Margin), RM (River Margin). The white diamond and the red rectangle represent the location of the study area within the Iberian Peninsula (upper right corner) and the eastern end of the Ria Formosa (lower right corner). Images source: Google Earth, Airbus, April 2020.

The long-term dynamics of inlets and feedbacks with the barrier (and backbarrier) morphologies remain largely unexplored^17^. Therefore, effects of inlet opening or relocation that were not accounted for during the planning phase will continue to be observed in the future. The main objective of this work is to analyse the direct and indirect morphological consequences of an inlet opening in the Ria Formosa, southern Portugal (Fig. 1), and to identify the cascading effects of this opening. Possible challenges for the future coastal management of the area are also discussed.

## Results

### Inlet opening and morphological changes observed

**Figure 2:**
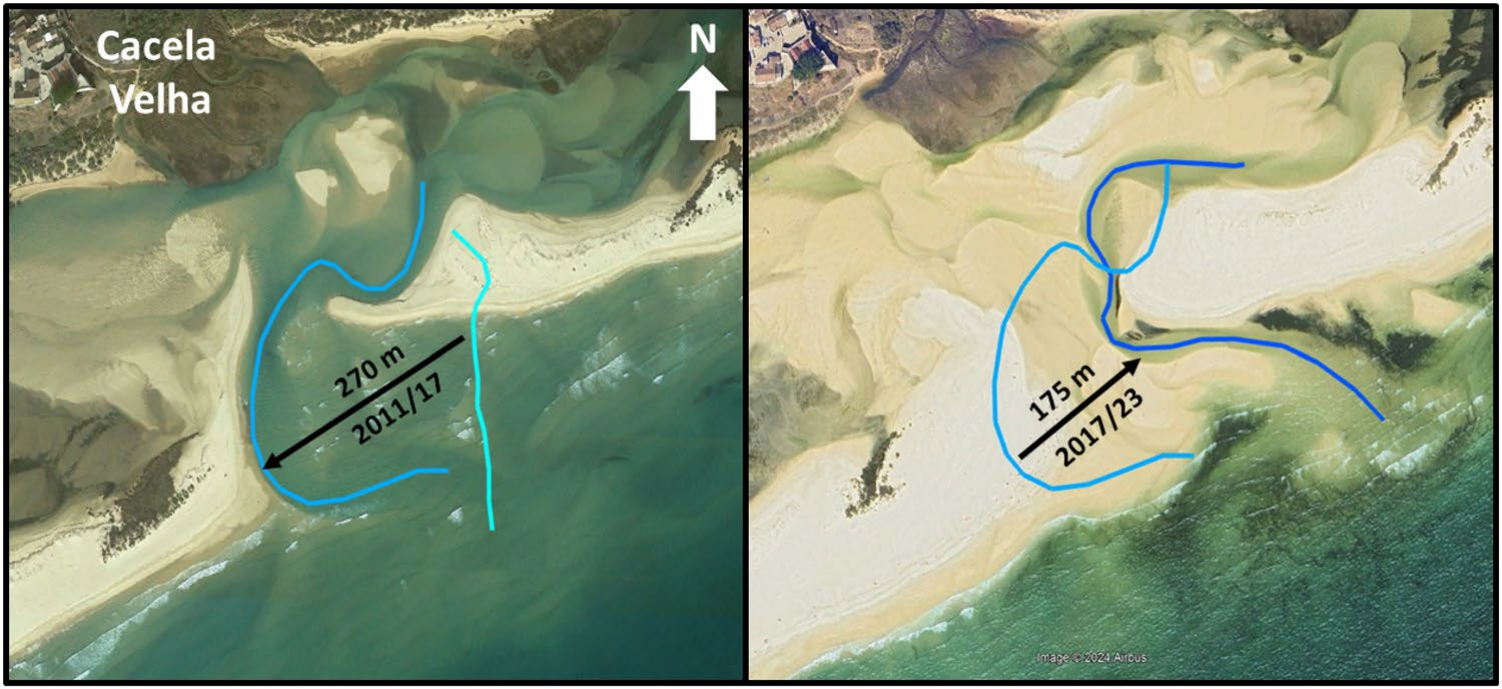
Displacement of the inlet channel (black arrows) for the periods 2011/2017 (left panel) and 2017/2023 (right panel). The lines indicate the channels’ position for the initial and final years. Images source: Google Earth, November 2017 (left panel) and Google Earth, Airbus, June 2023 (right panel).

**Figure 3:**
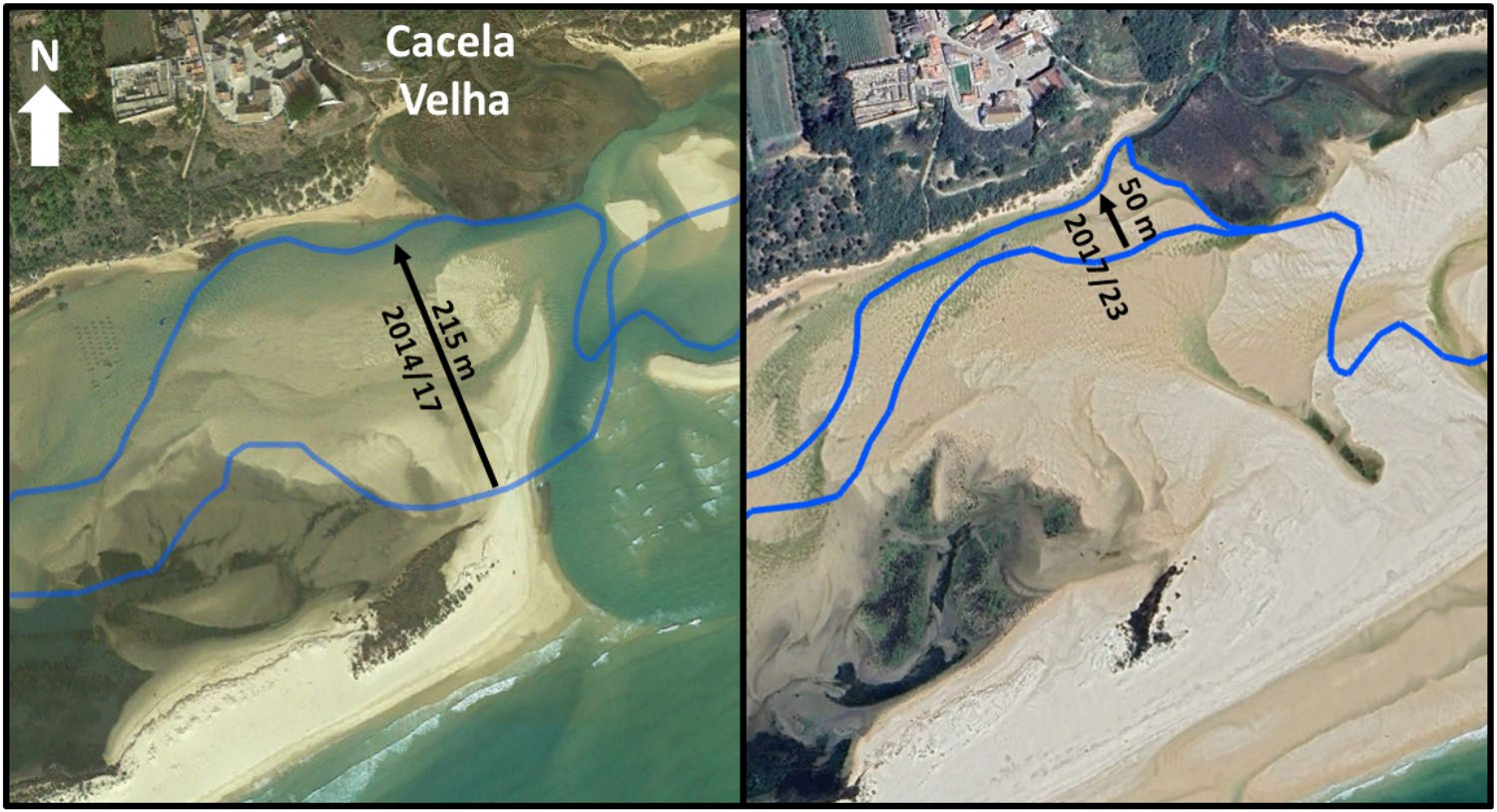
Displacement of the main tidal channel (black arrows) for the periods 2014/2017 (left panel) and 2017/2023 (right panel). The lines indicate the channels’ position for the initial and final years. Images source: Google Earth, November 2017 (left panel) and Google Earth, Airbus, April 2024 (right panel).

The artificial opening of the Cacela Inlet was completed in 2010, creating a main inlet channel oriented near North–South that quickly connected with the pre-existing lagoon tidal channel, which had been dredged in 2000. From 2011 to 2017, the main inlet channel meandered and migrated westward (see Fig. 2), but from 2017 to 2023, it migrated in the opposite direction, returning to a location near its original opening point. The opening of the Cacela Inlet resulted in the development of a flood delta on the lagoon side. This delta covered part of the existing Cacela River delta and a large portion of the formerly dredged lagoon tidal channel. Consequently, the tidal channel began to meander and shift position. Between 2014 and 2017, there was an important change in the channel’s behaviour. Its former location became completely silted up, and quickly migrated 215 m northward (see Fig. 3). Since 2017, the tidal channel has been connected to the beach at the lagoon margin near the Cacela Velha settlement and to the mouth of the Cacela River. Between 2017 and 2023, a further northward migration of about 50 m was observed in this tidal channel (Fig. 3).

**Figure 4:**
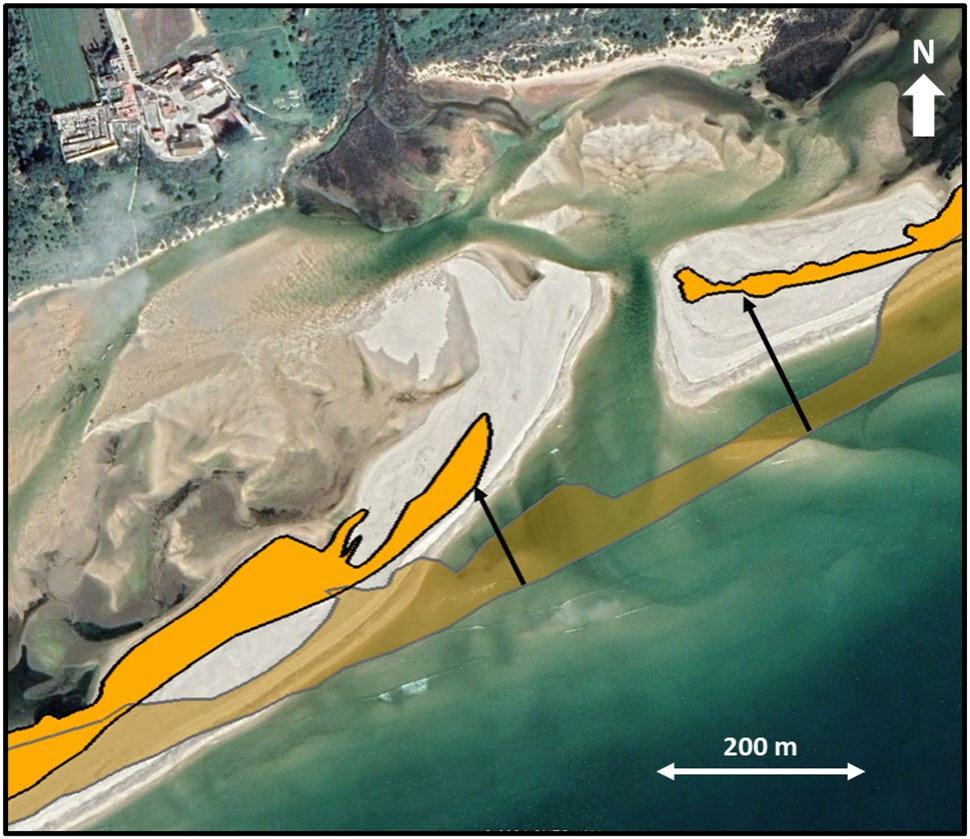
Oceanic coastline retreat and inland inlet migration between 2007 (before inlet opening; transparent orange) and 2021 (opaque orange). The orange areas in the image represent the vegetated (dune) area at the barriers. The arrows show the higher retreat near the inlet. Image source: Google Earth, May 2021.

Between 2007 and 2021, the oceanic coastline experienced a relevant retreat with averages of 3.1 m/year (to the west of the inlet opening position) and 9.5 m/year (to the east of the inlet opening position) (Fig. 4).These coastline retreat trends are exacerbated near the inlet due to inland migration of the sandy spits. The retreat rates on the 200 m section to the west and east of the inlet were 4.4 m/year and 11.6 m/year, respectively.

### Lagoon margin and river bank evolution

**Figure 5:**
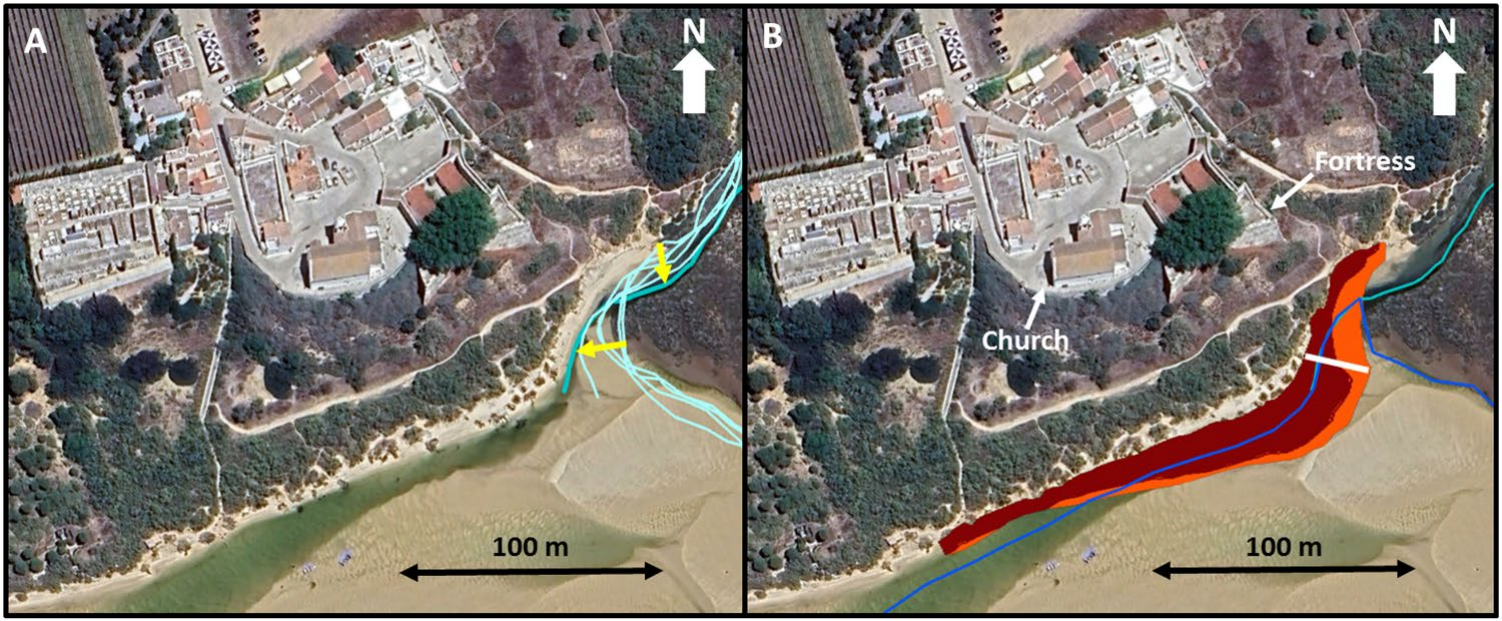
(**A**) Meandering of the terminal part of the Cacela River, between 2007 and 2023 (higher displacement values marked with yellow arrows). (**B**) Erosion of the river and lagoon margins for 2007/2017 (orange) and 2017/2023 (brown). The blue lines represent the centre of the tidal channel (dark blue) and of the Cacela River (light blue). The margins of the river (to the north) and the lagoon (to the west) are separated by the white line. Image source: Google Earth, Airbus, June 2023.

**Figure 6:**
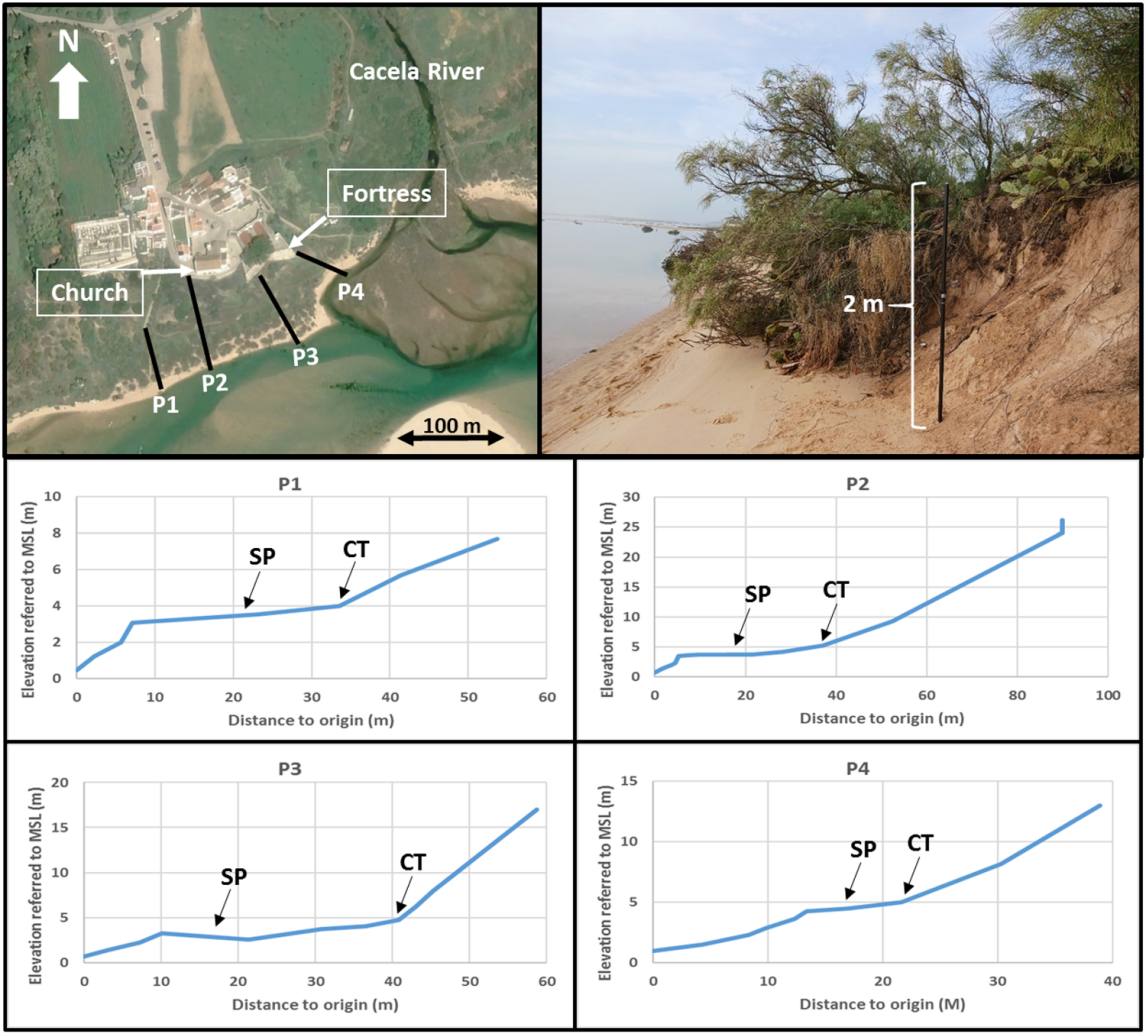
Location of the topographic profiles (P1 to P4, top left panel), evidence of strong retreat by scarp formation and consequent vegetation fall (top right panel), and topographic profiles from the lagoon (origin) to a mid or upper cliff position (lower panels). SP—Sandy platform, CT—Cliff toe. Aerial image source: Google Earth, Airbus, April 2020.

The contact between the Cacela River and the lagoon is defined by the southern end of a meander (Fig. 1). During the study period (2007/2023), the meandering process of the terminal part of the river continued with a 16 m southerly displacement of the northern part of the meander bend and a 21 m migration of the meander cut bank (Fig. 5A). The rapid northward shift of the lagoon’s tidal channel between 2014 and 2017 was responsible for its direct connection to the meander and thus to the mouth of the Cacela River. The river margin presented an average retreat of 0.9 m/year and 1.15 m/year, for the periods 2007/2017 and 2017/2023, respectively, with a minimum value of about 0.4 m/year for both periods, while the maximum values reached 1.3 m/year (2007/2017) and 2.1 m/year (2017/2023) (Table 1; Fig. 5B). The lagoon margin showed a smaller average retreat of 0.2 m/year for the period 2007/2017, with values ranging from a coastline advance of 0.2 m/year to a retreat of 0.7 m/year. This behaviour changed for the period 2017/2023, with an acceleration of almost 10 times in the average retreat rates, which reached almost 2 m/year, ranging between 0.8 m/year and 3.5 m/year (Table 1). Overall, the joint eroded area of the lagoon and river margins was 93 m^2^/year for the period 2007/2017, which increased to 412 m^2^/year for the period 2017/2023 (Fig. 5B). In March 2024, both the lagoon and river margins presented a narrow (< 15 m) and very steep (slopes from 0.21 to 0.37) sandy beach, which was connected to an upper vegetated sandy surface by an erosive scarp up to 2 m high (Fig. 6). This sandy surface separates the lagoon from the inactive Cacela cliff and has a variable width, ranging from 13.2 to 30. 8 m in the study area, with an average of about 25 m (Fig. 6).

**Table 1:** Coastline evolution, in m/year, for the periods 2007/2017 and 2017/2023, for each considered transect on the lagoon (LM) and river (RM) margins, and associated means.

	2007/2017	2017/2023
Lagoon		
LM1	− 0.06	− 1.03
LM2	− 0.17	− 0.77
LM3	+ 0.16	− 1.17
LM4	+ 0.09	− 0.92
LM5	− 0.26	− 0.90
LM6	− 0.32	− 1.33
LM7	− 0.34	− 1.70
LM8	− 0.31	− 2.20
LM9	− 0.24	− 2.57
LM10	− 0.09	− 2.97
LM11	− 0.22	− 2.88
LM12	− 0.37	− 3.53
LM13	− 0.69	− 3. .32
Mean	− 0.22	− 1.94
River		
RM1	− 1.29	− 2.12
RM2	− 1.31	− 1.37
RM3	− 1. .03	− 0.57
RM4	− 0.51	− 1.28
RM5	− 0.46	− 0.42
Mean	− 0.92	− 1.15

Negative values represent retreat and positive values represent advance. For the transects location see Supplementary Fig. 1.

## Discussion

**Figure 7:**
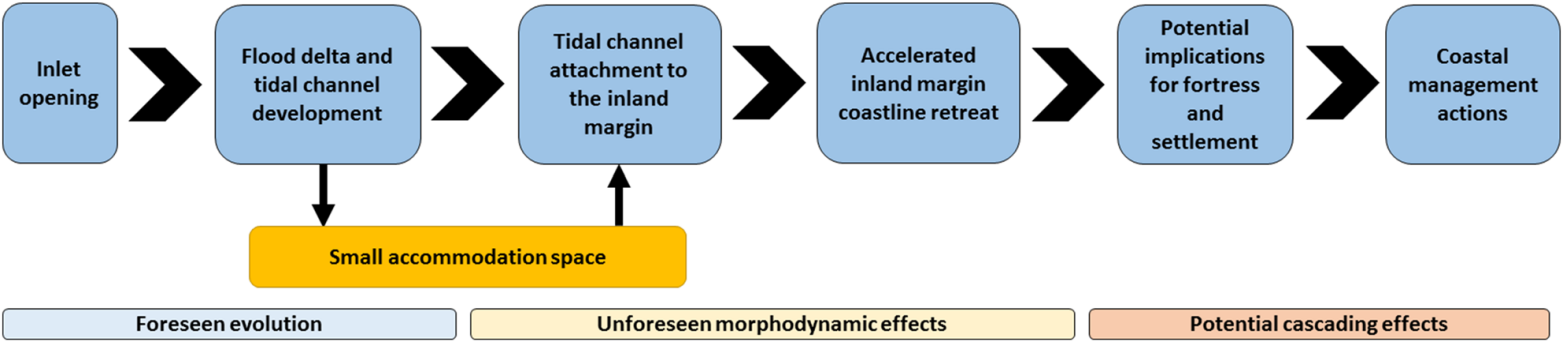
Overall sequence from the opening of the inlet to the final need for coastal management measures, including unforeseen morphodynamic effects and associated potential cascading effects.

After its opening in 2010, the new Cacela Inlet showed a high alongshore variability in the position of its main inlet channel, but also an overall stability of its mean position, with no alongshore migration trend for the period 2011/2023. This is in agreement with previous observations^18^ for a shorter analysis period (2015/2020). The observed retreat rates of the oceanic coastline near the inlet, ranging from 4 to almost 12 m/year, are also consistent with previously observed trends^18,22^, which report an inland migration of the inlet. As the inlet migrated northwards (inland), it also developed a large flood tidal delta and tidal channels. The existing accommodation space for the development of these morphologies was quite small, with a maximum lagoon width (in 2011) of only 400 m from the inlet mouth to the inland lagoon margin. As a result, the flood delta developed partially over the former river delta, reducing its total area and occupying the former tidal channels, which were dredged in 2000 and have remained in the same position since then. The formation of the flood delta and the silting up of the former tidal channel forced an inland shift of the main tidal channel, which in 2017 connected to the lagoon’s inland margin and the river mouth. The formation of new deltas and the morphological transformation of former deltas and sand-flats are well known consequences of inlet openings in Ria Formosa^19^. However, there are several other reported unforeseen effects of inlet openings on this barrier system, such as narrowing of adjacent inlets, channel migration or island growth^4,19,20^, with observed cascading effects such as damage and destruction of property^11,12^. The main unaccounted morphodynamic effect of the opening of this inlet in 2010 and the subsequent morphological evolution was observed after 2017, as a result of the inland shift of the main tidal channel. It corresponds to an extreme acceleration of the retreat of the inland lagoon margin from about 0.2 to 2 m/year (a tenfold increase), exceeding an already high retreat caused by the river meandering (about 1 m/year). Such drastic changes can occur when new inlets are opened, and are exacerbated when they are placed where none existed before^15^, as was the case. The effect of the tidal channel attachment to the river mouth was relatively small, increasing the retreat rate on the river margin by a factor of only 1.25. One consequence of the observed acceleration of the retreat rates is the erosion of the flat sandy surface that currently separates the old and inactive Cacela cliff from the lagoon. This flat sandy platform has an average width of about 25 m, with a minimum of about 13 m near the meander cut bank. This means that sometime between 2035 and 2040, the toe of the cliff will become active again, with a strong possibility of potential impacts on the safety of the fortress and the existing settlement of Cacela Velha. This represents an unforeseen cascade effect, 25–30 years after the opening of the inlet (Fig. 7).

To avoid the instability of this legally protected area for its cultural and historical value, several coastal management interventions will be required. These could include dredging the migrated tidal channel to an inner lagoon position (similar to the one existing in 2010) and using the dredged sediments to fill in the existing inner tidal channel, thereby increasing the width of the beach at the lagoon margin. These measures would most likely be temporary, as the post-intervention coastal evolution would include the inland migration of the tidal channel until it reaches its current position in a few years/decade. In addition, a management intervention would also be required at the river meander, by promoting an artificial meander cut-off and a realignment of the river, with the main channel flowing in a straight line to the lagoon, creating a small (probably temporary) oxbow lake.

The indirect and unforeseen effects of the opening of the Cacela Inlet have been felt mainly from the 7 years after the opening until now, but will continue for the next decades if no action is taken. These effects (accelerated retreat of the lagoon and river margins) will potentially lead to a cascade effect, the instability of the Cacela cliff, which may threaten the culturally and historically valuable settlement of Cacela Velha, including the existing fortress. The analysis carried out reinforces the need for a more comprehensive long-term analysis when implementing coastal management measures in dynamic environments such as inlets, which must always be linked to medium to long-term coastal monitoring.

## Methods

The analysis of the coastal evolution, including the evolution of the oceanic coastline, the main inlet channel, the main lagoon tidal channel, the lagoon and river margins, and the meander of the Cacela River, was carried out by analysing aerial images. The images used were obtained from Google Earth Pro and analysed at the highest possible visualisation resolution. The pixel size of the images used varies from year to year (from about 20 to 60 cm), which also varies the resolution of the morphological analysis. All distances and areas have been calculated using Google Earth Pro tools (e.g. polygon areas, ruler). The dates chosen for the analysis, based on the quality of the available images, were June 2007 (before the opening of the inlet), May 2011 (after the opening), October 2014, November 2017, April 2020, March 2021 and June 2023. The available imagery from June 2023 covered only the western part of the study area and was used for the analysis of the tidal channels and coastline evolution on the lagoon and river margins, but not for coastline evolution on the ocean side. Images from 2011 were mostly used for qualitative assessment or large-scale analysis due to the lower overall image quality.

The assessment of the evolution of the oceanic coastline was carried out by comparing the coastline between 2007 and 2021. The vegetated dune/beach contact was defined as representative of the coastline, with embryo and sparsely vegetated dunes being considered as part of the dune system. A total of 30 cross-shore transepts spaced 50 m apart (20 to the west and 10 to the east of the inlet) were used to define the overall coastline evolution.

The main inlet channel was marked for all images analysed, using the deepest (darkest) part of the channel as a reference. Its alongshore movement was assessed by comparing the displacement of the intersection of the inlet channel with a fixed reference line crossing the inlet and connecting the two spits (see black arrows in Fig. 2 as an example). The main tidal channel of the lagoon was marked on all images analysed using the deepest (darkest) part of the channel as a reference. At low tide and in the absence of water, the most incised (lower) morphology was considered to correspond to the centre of the lagoon tidal channel (see the blue lines in Fig. 3 as an example). In some cases the channel presented a bifurcation and two channels were marked. The position of the Cacela River channel was also marked. The evolution of these morphologies was mostly assessed qualitatively.

The coastlines of the lagoon and river margins were marked by the contact between the bare sand of the beach and the vegetation line. In several cases it was possible to observe that a beach scarp existed and that part of the vegetation was already eroded and lying on the beach (Fig. 6, top right panel). In these cases, the existing beach scarp was marked as corresponding to the coastline. The 2023 coastline was used as a reference line against which the others were compared, using transepts at 25 m intervals. The river margin was included in the analysis because of its direct connection to the lagoon margin and its proximity to the Cacela cliff toe and the existing fortress. The coastal evolution of the lagoon and river margins in front of the Cacela settlement was analysed for two different periods: (a) 2007/2017, representing the evolution from before the opening of the inlet until the connection of the tidal channel to the lagoon margin; (b) 2017/2023, after the connection of the tidal channel to the lagoon margin and to the river mouth. In order to separate the evolution dominated by the meandering (fluvial) process and the displacement of tidal channel (lagoon), the position of the river mouth in 2017 was used to define a boundary between the processes dominated by the river (to the north) and the lagoon (to the west), resulting in a total of 13 transepts analysed for the lagoon margin and five for the river margin. For the transects location see Supplementary Fig. 1.

The georeferencing error of the images was analysed using six control points located near the lagoon margin, for the images of 2023, 2021, 2020, 2017 and 2014, and four control points for 2007. The June 2023 images were used as a reference to calculate the differences. The root mean square errors ranged between 0.8 m (2021 and 2020) and 4.9 m (2007), with only the 2007 and 2014 images having values greater than 1 m.

In order to complete the information and to obtain a better morphological description of the inner lagoon and the river margin and their transition to the inactive Cacela cliff, four topographic profiles were surveyed in February 2024, using a GNSS-RTK system (Fig. 6). The profiles were carried out in a point-by-point mode due to the difficulty of accessing both the heavily vegetated (often with prickly pear cactus, *Opuntia ficus-indica*) sandy platform and the steep cliff.

## Study area

The Ria Formosa lagoon and barrier system (Fig. 1) is subject to semidiurnal tides with a maximum tidal range of about 3.5 m. The mean annual significant offshore wave height is about 1 m with an average peak period of 8.2 s^21^. The eastern flank of the system is relatively sheltered from the W-SW dominant waves and storms, while being exposed to the less frequent E-SE incident waves. The net littoral drift and longshore transport is from west to east with variable magnitude along the Ria Formosa system^19^.

Cacela Inlet is the easternmost inlet of the Ria Formosa barrier island system, separating the Cacela Peninsula (to the east) from Cabanas Island (to the west) (Fig. 1). The boundary between these two barriers was previously (before 2010) defined by the former Lacém Inlet, the smallest of the entire system^3^, which migrated from west to east^13^. The Lacém Inlet was characterised by high variability in width and migration rates, with a tendency to narrow towards the east^22^. The western part of the former Cacela Peninsula was eroded by the migration of the Lacém Inlet and barrier breaches^23^ and was subject to several human interventions such as inlet and channel dredging and beach/dune nourishment^22,24,25^. In 2010, a small inlet (the new Cacela Inlet) was artificially opened near the eastern tip of the system, in a position east of any known position since the 1940s^19^, altering the relative positions and morphologies of Cabanas Island and Cacela Peninsula. Currently, the Cacela Peninsula is a narrow (< 150 m wide) and small (about 1.5 km long) sand spit covered by vegetated dunes^19^. The main channel of the Cacela Inlet showed (between September 2015 and September 2020) a high variability in its position, with alongshore shifts of about ± 100 m with respect to its mean position, but without an alongshore migration trend. On average, it remained approximately at its artificial opening position, but the inlet showed a slight trend to migrate cross-shore towards the lagoon^18^. The former Lacém Inlet (and by analogy the current Cacela Inlet) drained a quasi-independent sub-embayment located at the eastern end of the Ria Formosa lagoon^26,27^.

**Figure 8:**
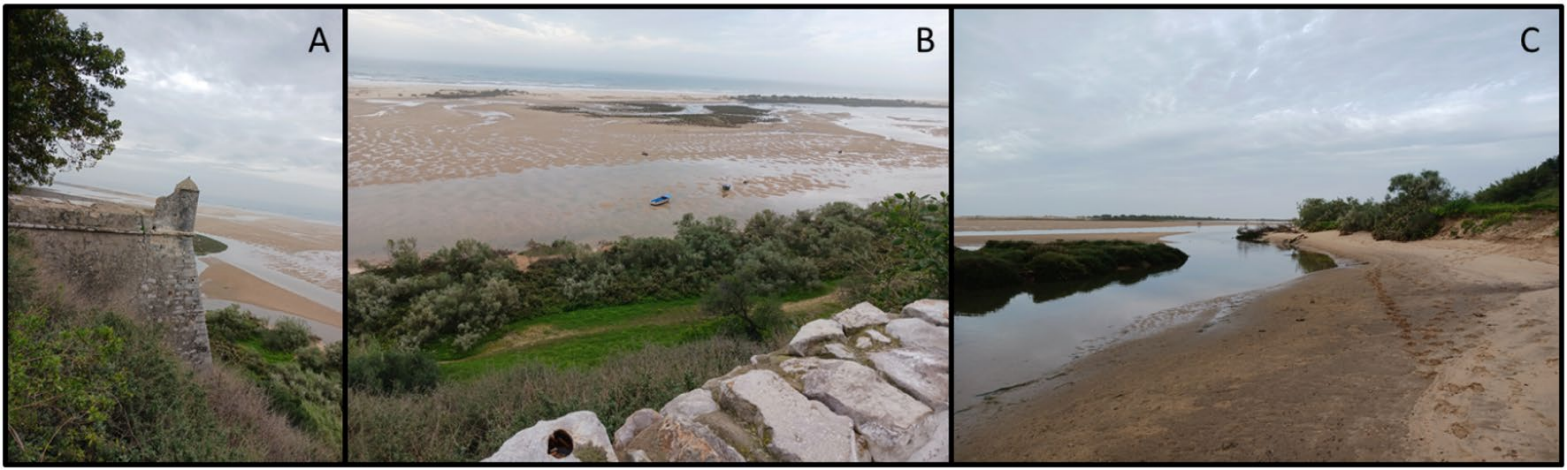
(**A**) The western bastion of the Cacela fortress, located in the middle of the cliff. (**B**) View from the church of Cacela Velha to the south, over the cliff, the densely vegetated sandy surface and the lagoon (at low tide) with the tidal channel connected to the lagoon’s inland margin. (**C**) The river margin and the connection of the Cacela River to the tidal channel.

Cacela Inlet and the Cacela Peninsula take their names from the neighbouring settlement of Cacela Velha, which is located just inland from the lagoon, on the top of an inactive cliff and on the right bank of the Cacela River (Ribeira de Cacela). The historic centre of Cacela Velha has been a protected area since 1996, due to its national and public interest^28^. The historic nucleus of the Cacela Velha settlement includes a sixteenth-century church and a medieval fortress (Figs. 4 and 5), which has undergone several modifications since its construction^28^. These two important structures are located on the cliff top and, in the case of the fortress, the bastions are located in the middle of the cliff, less than 50 m from the lagoon and river margins (Fig. 8). These margins consist of a sandy beach bordered by densely vegetated sandy areas that connect inland to the inactive cliff.

The Cacela River, known for its famous outcrop that serves as the reference section for the geological Cacela Formation and as a key section for the analysis of the molluscan biodiversity of the Late Miocene^29^, marks the eastern boundary of the Cacela Velha settlement. The Cacela River, actually a small ephemeral stream, communicates with the lagoon through a meander (Fig. 8) located near the eastern bastion of the Cacela Fortress. In front of its mouth, inside the lagoon, a small delta has developed, covered with salt marshes, influenced by the tides and tidal currents (Fig. 1).

### Supplementary Information

Supplementary Figure 1.

## Data Availability

The data used in this study are available on request from the author (Óscar Ferreira, oferreir@ualg.pt).
