# Author Correction: Unforeseen cascading effects of an inlet opening

**DOI:** 10.1038/s41598-024-67608-3

**Published:** 2024-07-16

**Authors:** Óscar Ferreira

**Affiliations:** CIMA‑Centre for Marine and Environmental Research, FCT, University of Algarve, Faro, Portugal

Correction to: *Scientific Reports* 10.1038/s41598-024-63467-0, published online 11 June 2024

The original version of this Article contained an error in the Abstract.

“Between 2025 and 2030, the currently inactive Cacela cliff is likely to become active again, posing a potential risk of damage to a medieval fortress and the existing settlement of Cacela Velha, an unforeseen cascading effect of the opening of the inlet.”

now reads:

“Between 2035 and 2040, the currently inactive Cacela cliff is likely to become active again, posing a potential risk of damage to a medieval fortress and the existing settlement of Cacela Velha, an unforeseen cascading effect of the opening of the inlet.”

In addition, in the Discussion section,

“This means that sometime between 2025 and 2030, the toe of the cliff will become active again, with a strong possibility of potential impacts on the safety of the fortress and the existing settlement of Cacela Velha. This represents an unforeseen cascade effect, 15–20 years after the opening of the inlet (Fig. 7)”

now reads:

“This means that sometime between 2035 and 2040, the toe of the cliff will become active again, with a strong possibility of potential impacts on the safety of the fortress and the existing settlement of Cacela Velha. This represents an unforeseen cascade effect, 25–30 years after the opening of the inlet (Fig. 7)”

The original Article has been corrected.

