# Supplementary material for: Unforeseen cascading effects of an inlet opening
Source: Sci Rep. 2024 Jun 11;14:13421. doi: 10.1038/s41598-024-63467-0 (PMC11166913; doi:10.1038/s41598-024-63467-0)
Supplement: Supplementary file 1 — Supplementary Figure 1. [file 41598_2024_63467_MOESM1_ESM.docx]

Supplementary Information


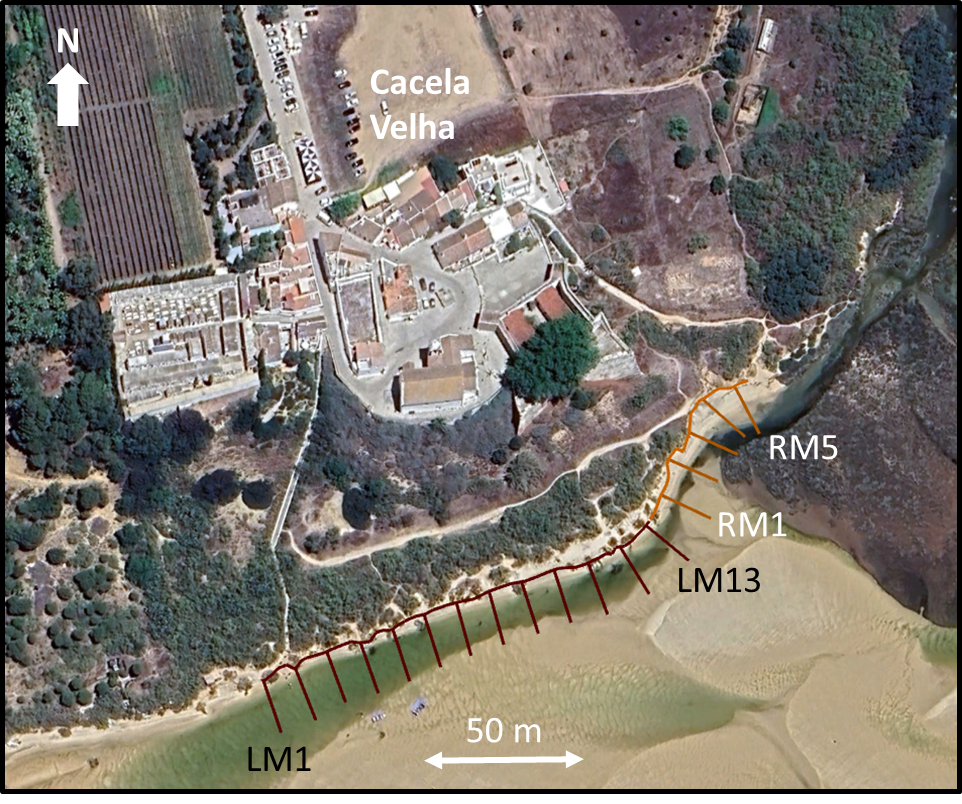


Supplementary Figure 1. Location of the transects used to determine the coastal evolution, with their origin at the 2023 coastline. LM1 to LM13 refer to the transects used for the lagoon margin, while RM1 to RM5 refer to the transects used for the river margin. Image from Google Earth, June 2023.
